# Individual isotoxic radiation dose escalation based on V20 and advanced technologies benefits unresectable stage III non-small cell lung cancer patients treated with concurrent chemoradiotherapy: long term follow-up

**DOI:** 10.18632/oncotarget.16288

**Published:** 2017-03-16

**Authors:** Ming Liu, Zhongtang Wang, Tao Zhou, Antang Zhou, Qian Zhao, Hongsheng Li, Hongfu Sun, Wei Huang, BaoSheng Li

**Affiliations:** ^1^ School of Medicine and Life Sciences, University of Jinan-Shandong Academy of Medical Sciences, Jinan, Shandong, P.R. China; ^2^ Department of Radiation Oncology, Shandong Cancer Hospital Affiliated to Shandong University, Shandong Academy of Medical Sciences, Jinan, Shandong, P.R. China; ^3^ Department of General Surgery, Yanggu People’s Hospital, Liaocheng, Shandong, P.R. China; ^4^ Engineering Research Center for Medical Imaging and Radiation Therapy of Shandong Province, Jinan, Shandong, P.R. China

**Keywords:** non-small cell lung cancer, chemoradiotherapy, lung V20, Individual isotoxic dose escalation, advanced radiotherapy technologies

## Abstract

Under the assumption that the highest therapeutic ratio could be achieved by increasing the total tumor dose (TTD) to the limits of normal tissues, the phase I trial was conducted in patients with unresectable stage III non-small cell lung cancer treated with concurrent chemoradiotherapy, to determine the feasibility and effects of individual isotoxic radiation dose escalation based on bilateral lung V20 and advanced technologies. Consecutive eligible patients were assigned to cohorts of eight. V20 of each cohort was increased from 27% to 30%, 33%, 35%, 37%, and so on. The criterion for cessation of dose escalation was defined as ≥ 2 patients in each cohort experienced dose limiting toxicity. Isotoxic dose escalation was based on V20, functional imaging was used to improve the accuracy of radiotherapy. To test the power of escalation dose, patients with TTD over 66 Gy were assigned to the higher dose group (HD), while the others to the standard dose one (SD). In result, the recommended value of V20 was 35%. For all patients, follow-up ranged from 1 to 112 months, median overall and progression free survivals were 25.0 and 13.0 months, respectively. The 1-, 3-, 5- and 8-year overall survival (OS) rates were 72.5%, 22.5%, 17.5%, and 10.0%, respectively. Especially, the OS and local recurrence-free survival of patients in HD group were significantly longer than those in SD one (*P*=0.035, P=0.007, respectively) without increasing severe toxicity. Thus, individual isotoxic dose escalation based on V20 with advanced technologies was feasible and effective.

## INTRODUCTION

Non-small cell lung cancer (NSCLC) remains one of the most common and fatal malignancies, and approximately 35% of patients with NSCLC present with locally advanced disease [1]. Concurrent chemoradiotherapy (CCRT) has been established as the standard care for unresectable locally advanced NSCLC (LANSCLC), however, the prognosis is still unsatisfactory [2, 3]. Radiation dose escalation has been shown to be able to improve local control and overall survival (OS) of patients with LANSCLC in several studies [4–9]. However, the Radiation Therapy Oncology Group (RTOG) 0617 trial indicated that higher radiation dose of 74 Gy did not produce an OS benefit compared with the standard dose of 60 Gy, and the most possible explanation of this result might be the greater toxicity on the normal tissue [10]. Thus, the safety and efficacy of radiation dose escalation in the setting of CCRT for LANSCLC remains uncertain, and personalised isotoxic radiation dose escalation ensuring the safety of the organs at risk (OARs) may be a potential solution [11–12].

Lots of studies on radiotherapy for patients with LANSCLC focused on new radiation approaches. Modern advanced techniques such as 18F-fluorodeoxyglucose positron emission tomography/computed tomography (18F-FDG PET-CT) and intensity-modulated radiation therapy (IMRT) have substantially enhanced the accuracy of the radiotherapy delivery through improved target conformality [13–15]. The identification of functional lung using lung perfusion information obtained from single-photon emission computed tomography (SPECT), coupled with IMRT had the potential to reduce the pulmonary toxicity [16–21]. Shrinking field during treatment course might be feasible to spare more normal tissues [22]. Accelerated and hyperfractionated radiotherapy schedules resulting in reduced overall treatment time and less tumor repopulation have been shown to improve outcomes in LANSCLC compared to conventional fractionation [23–24]. These approaches, either alone or in combination, could enable radiation dose escalation without increasing the risk of toxicity, and help to improve local-regional control and OS in LANSCLC.

In addition, bilateral lung V20 (volume of the whole lung receiving ≥ 20 Gy) is one of the most important predictors for radiation-induced lung toxicity and has been commonly used as a constraint for dose escalation. Thus, in this study, we investigated the feasibility and effects of individual isotoxic radiation dose escalation in patients with unresectable stage III NSCLC treated with CCRT based on V20, SPECT, 18F-FDG PET-CT, and late-course accelerated hyperfractionated (LCAHF) IMRT.

## RESULTS

### Patient characteristics

Thirty-seven males and three females were enrolled, with a median age of 62 years (range, from 34 to 72 years). The patient characteristics were presented in Table 1. Stage distribution was as following: IIIa 42.5% (*n* = 17), IIIb 57.5% (*n* = 23). Most patients had squamous carcinoma (60.0%). No perfusion defects were observed in 3 patients (7.5%), while grade 1 perfusion defects were observed in 13 patients (32.5%), grade 2 in 15 patients (37.5%), and grade 3 in 9 patients (22.5%). All patients completed the study protocol treatment, however, 3 patients’ treatments were suspended for 7 to 14 days because of severe hematological toxicity.

**Table 1:** Patient characteristics in this phase I study

Characteristics	Cohort 1	Cohort 2	Cohort 3	Cohort 4	Cohort 5
Gender, n (%) Male Female					
	7 (87.5)	7 (87.5)	8 (100)	7 (87.5)	8 (100)
	1 (12.5)	1 (12.5)	0 (0)	1 (12.5)	0 (0)
Age (years), Median (range)	66 (43–71)	56 (40–71)	57 (34–72)	61(51–72)	62(48–71)
Histolgy, n (%) Squamous carcinoma Adenocarcinoma Large cell carcinoma					
	6 (75.0)	7 (87.5)	5 (50.0)	2 (25.0)	4 (50.0)
	2 (25.0)	1 (12.5)	2 (12.5)	4 (50.0)	1 (12.5)
	0 (0)	0 (0)	1 (37.5)	2 (25.0)	3 (37.5)
Stage, n (%)					
IIIa	7 (87.5)	3 (37.5)	3 (37.5)	2 (25.0)	2 (25.0)
IIIb	1 (12.5)	5 (62.5)	5 (62.5)	6 (75.0)	6 (75.0)
Tumor localization, n (%) Upper lobes Lower lobes Hilar areas					
	4 (50.0)	5 (62.5)	4 (50.0)	4 (50.0)	3 (37.5)
	3 (37.5)	2 (25.0)	2 (25.0)	2 (25.0)	2 (25.0)
	1 (12.5)	1 (12.5)	2 (25.0)	2 (25.0)	3 (37.5)
Performance status, n (%) 0 1					
	5 (62.5)	1 (12.5)	3 (37.5)	4 (50.0)	4 (50.0)
	3 (37.5)	7 (87.5)	5 (62.5)	4 (50.0)	4 (50.0)
Perfusion deficit grade, n (%) 0 1 2 3					
	0 (0)	1 (12.5)	1 (12.5)	1 (12.5)	0 (0)
	1 (12.5)	3 (37.5)	3 (37.5)	3 (37.5)	3 (37.5)
	3 (37.5)	3 (37.5)	3 (37.5)	2 (25.0)	4 (50.0)
	4 (50.0)	1 (12.5)	1 (12.5)	2 (25.0)	1 (12.5)
Total tumor dose, n(%)					
≤66 Gy	5 (62.5)	4(50.0)	7(87.5)	1(12.5)	2(25.0)
>66 Gy	3 (37.5)	4(50.0)	1(12.5)	7(87.5)	6(75.0)

Abbreviations: n = number.

### Radiotherapy dose escalation and maximum tolerated value

The first and second cohorts (8 patients each) were treated with a mean radiotherapy dose of 62.2 (95% CI, 59.8-64.6 Gy) and 62.9 Gy (95% CI, 57.9-67.8 Gy), respectively. None of these patients had radiation pneumonitis (RTP) or radiation esophagitis (RTE). In the third and fourth cohorts, mean radiotherapy doses were 63.6 (95% CI, 57.5-69.7 Gy) and 68.3 Gy (95% CI, 60.9-75.6 Gy), respectively, and each cohort had 1 patient experienced grade 3 chronic RTP. Patients in the fifth cohort were treated with a mean radiotherapy dose of 71.3 Gy (95% CI, 66.7-75.8 Gy), and 2 patients (25.0%) developed grade 3 acute RTP while 1 patient (12.5%) developed grade 3 chronic RTP, thus reaching the maximum tolerated value (MTV). Therefore, a V20 equal to 37% was the MTV according to the study design, and the trial was closed to accrual. Thus, 35% was recommended as V20 value in isotoxic radiotherapy. Total tumor dose (TTD) was escalated along with the increasing of V20 in the cohorts, especially in the last 2 cohorts. Finally, 19 patients were assigned to the standard dose (SD) group (mean, 59.4 Gy, range, 51.2-65.2 Gy), while 21 patients to the higher dose (HD) one (mean, 71.3 Gy, range, 68.0-79.2 Gy).

### Toxicity

Toxicity of the patients were summarized in Table 2. Most toxicities, including diarrhea, skin problems, weight loss, and hemoptysis were common but mild. Thirty percent of all patients had grade 2 toxicities with asthenia, vomiting, and esophagitis that were rapidly resolved after the start of supportive care, antiemetic therapy, and steroids, respectively. Hematology toxicity was not frequent, but 5 patients with grade 3 and 1 patient with grade 4 hematological toxicities were prescribed granulocyte colony-stimulating factor during treatment. Ten of forty patients (25.0%) experienced severe acute toxicity (≥ grade 3) while three patients (7.5%) experienced severe late toxicity (≥ grade 3). No statistically significant difference was found in overall severe toxic events (≥ grade 3) between the SD group and HD group (4 of 19 patients [21.1%] vs. 7 of 21 patients [33.3%], *P* = 0.488), or in severe pulmonary toxicity between the two groups (2 of 19 patients [10.5%] vs. 3 of 21 patients [14.3%], *P* = 1.00).

**Table 2:** Toxicity during this phase I study as scored according to CTCAE v3

Toxicity, n	Grade 0	Grade 1	Grade 2	Grade 3	Grade 4
Asthenia	13	17	10	0	0
Vomiting	9	16	12	3	0
Esophagitis Acute Chronic					
	23	8	9	0	0
	29	7	4	0	0
Diarrhea	36	4	0	0	0
Skin	15	25	0	0	0
Lung Acute Chronic					
	20	10	8	2	0
	25	8	4	3	0
Weight loss	9	29	2	0	0
Pain	13	8	11	8	0
Hemoptysis	29	10	1	0	0
Hematology Erythrocyte Leukocyte Platelet					
	26	9	5	0	0
	11	12	11	5	1
	25	11	3	0	1

Abbreviations: CTCAE= Common Terminology Criteria for Adverse Events, n = number.

### Tumor response, overall survival, local recurrence free survival and progression free survival

Among the 40 patients, 7 achieved complete response, 25 partial response, and 8 stable disease after treatment. The overall response rate was 80%. There were no statistically significant differences in response rates between SD and HD groups (84.2% vs. 76.2%, *P* = 0.698), IIIa and IIIb groups (88.2% vs. 73.9%, *P* = 0.428).

**Figure 1:**
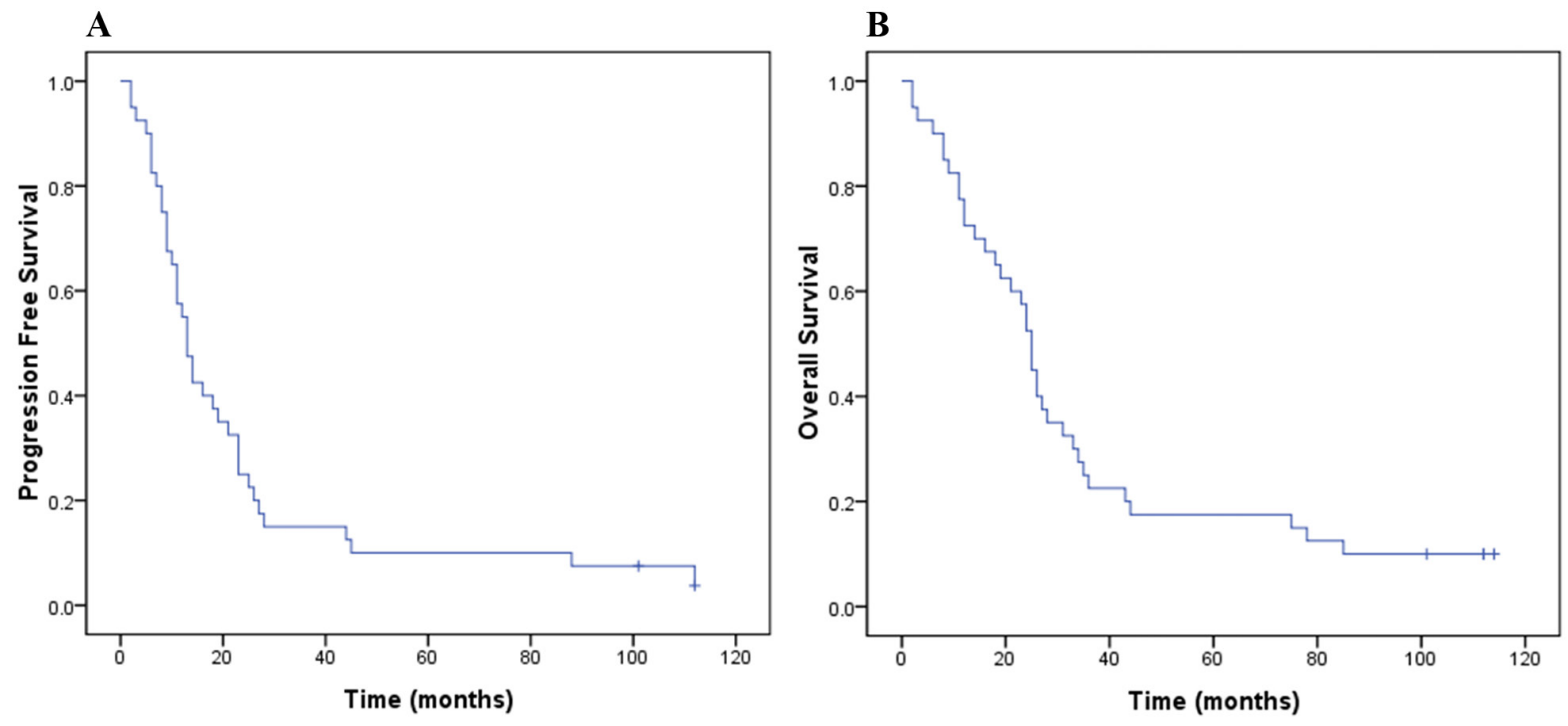
PFS in months for all patients (**A**), and OS for all patients (**B**).

By the last follow-up of Apr. 2016, follow-up for all patients ranged from 1 to 112 months (median, 22.5 months) with survival patients from 99 to 112 months. Median progression free survival (PFS) was 13.0 months (95% CI, 10.5-15.5 months; Figure 1A). Twenty-two patients (55%) developed recurrence within the target volume, while sixteen (40%) patients experienced distant metastases at the following sites: brain (4 patients), lung (5 patients), bone (4 patients), pleura (3 patients), non-regional lymph node (1 patient) and adrenal gland (1 patient). Thirty-six death events had occurred, including 18 in the SD group and 18 in the HD group. They all died of cancer related reasons except one patient died of heart attack. Median OS was 25.0 months (95% CI, 22.5-27.5 months), with 1-, 3-, 5- and 8-year OS rates of 72.5%, 22.5%, 17.5%, and 10.0%, respectively (Figure 1B).

### Uni- and multi-variate analysis survival

**Figure 2:**
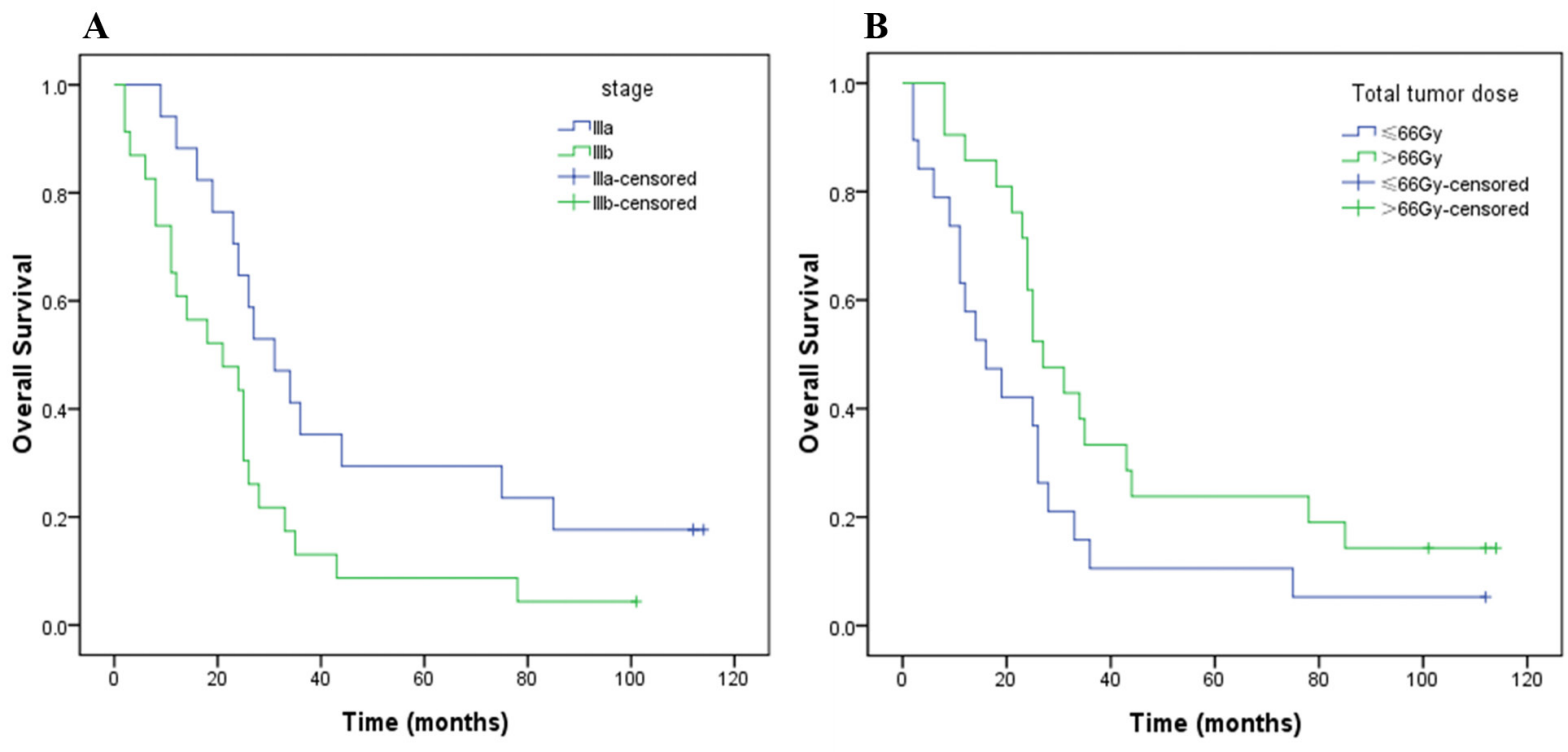
Kaplan–Meier OS curves for tumor stage (**A**) and total tumor dose (**B**).

**Figure 3:**
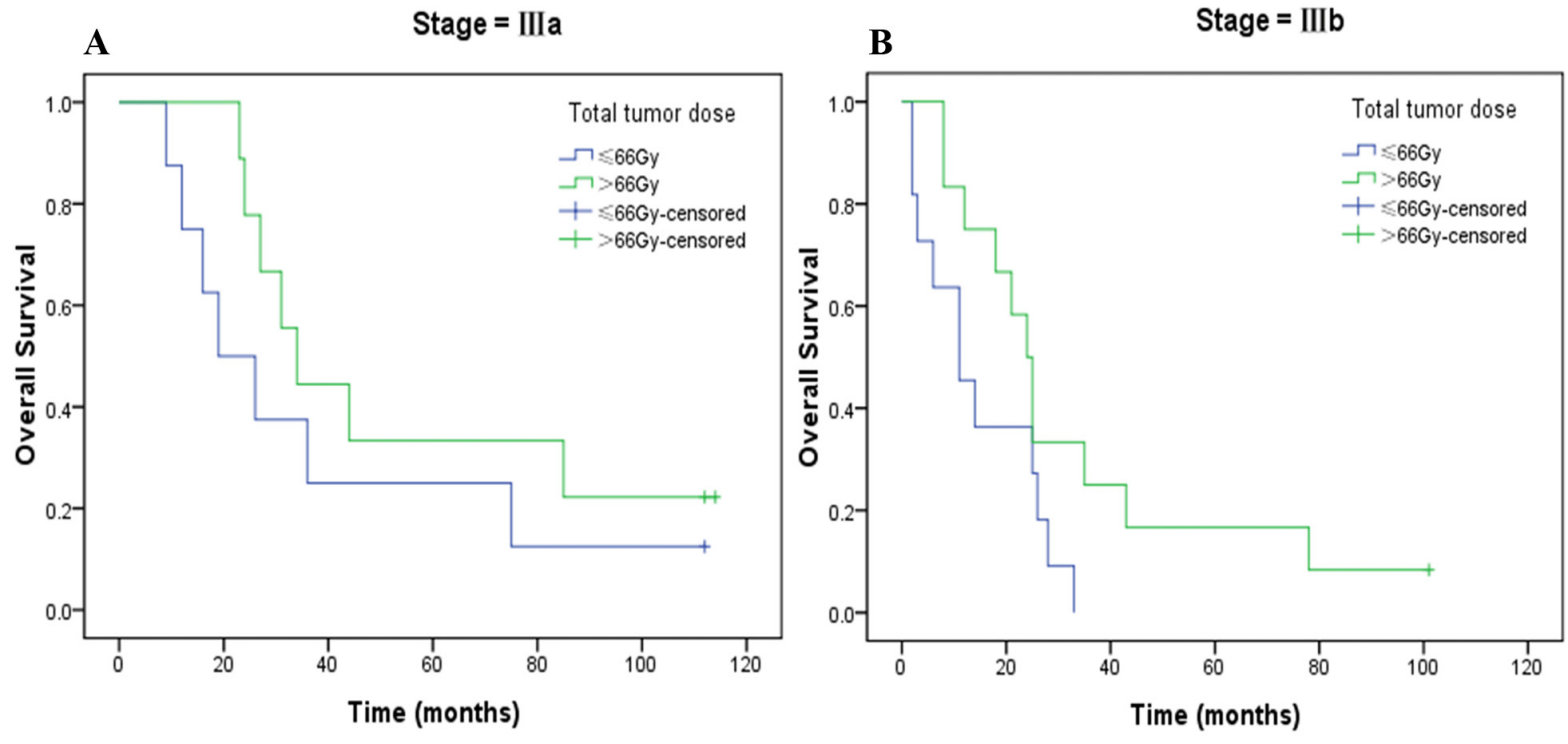
Kaplan–Meier OS curves for total tumor dose stratified by tumor stage (**A**, stage IIIa; **B**, stage IIIb).

**Figure 4:**
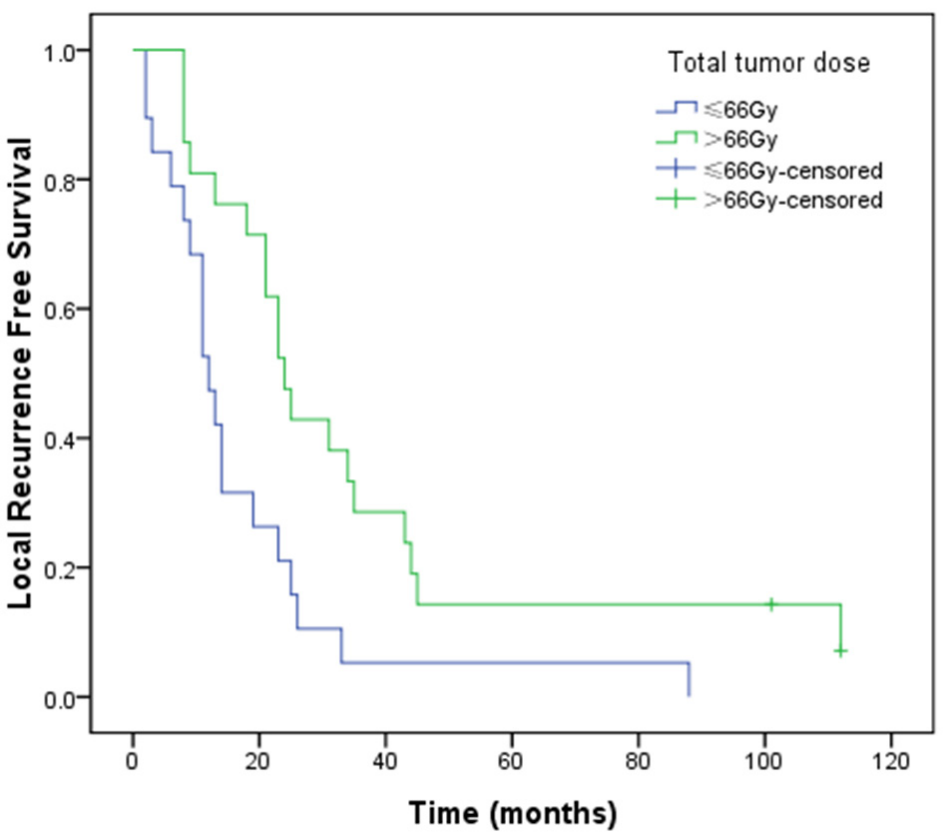
Kaplan–Meier LRFS curves for total tumor dose

On univariate analysis, patients of stage IIIa achieved a longer median OS than those of stage IIIb (31 vs. 21 months, *P* = 0.029; Figure 2A), and there was a trend that patients in HD group achieved a longer median OS than those in SD one (27 vs. 16 months, *P*= 0.053; Figure 2B). There was no significant correlation between stage and TTD in the chi-square test (*P* = 1.000). Noteworthily, in order to eliminate the effects of stage, univariate survival analysis was repeated testing TTD stratified by stage. The result indicated that patients in HD group achieved a significantly longer median OS than those in SD one (stage IIIa, 34 vs. 19 months; stage IIIb, 24 vs. 11 months; *P* = 0.035, Figure 3). Local recurrence free survival (LRFS) of HD group was also better than that of SD one (median, 24 vs. 12 months, *P* = 0.007; Figure 4). The other factors were not correlated with OS or LRFS, and all factors investigated were not correlated with PFS (Table 3). On multivariate analysis an early stage (*P* = 0.020) and HD (*P* = 0.033) remained independent factors for a better OS.

**Table 3:** Univariate and multivariate analysis for OS, PFS and LRFS

Factors	OS		PFS	LRFS
	Univariate	Multivariate	Univariate	Univariate
TTD	0.035*	0.033	0.144	0.007
Stage	0.029	0.020	0.084	0.261
Age	0.529	NA	0.980	0.684
Pathology	0.645	NA	0.262	0.926
V20	0.130	NA	0.131	0.353
Lung perfusion deficit	0.307	NA	0.302	0.444
Short-term effects	0.550	NA	0.443	0.466
Toxicity	0.475	NA	0.126	0.366
SUVmax	0.280	NA	0.624	0.417
SUVmean	0.291	NA	0.508	0.472

Abbreviations: OS= overall survival, PFS= progression free survival, LRFS= local recurrence free survival, TTD= total tumor dose, NA= not analysed, V20= volume of the whole lung receiving ≥ 20 Gy, SUVmax= maximal standard uptake value, SUVmean= mean standard uptake value.

* Result of univariate analysis stratified by stage.

## DISCUSSION

The recognition of cancer heterogeneity has driven us away from the ‘one size fits all’ approach and has allowed tailoring of treatment to individualised patient-tumor characteristics. However, the current radiotherapy is still prescribed at fixed doses (typically 60-66 Gy) for all patients with LANSCLC, and the prognosis is unsatisfactory [25]. Many researchers hold the idea that radiation dose escalation might improve local control and OS of patients with stage III NSCLC [26–29], but the accompanying increased radiation-related toxicity has limited its application. In addition, the outcome of the phase III RTOG 0617 trial was disappointing, in which higher radiation dose of 74 Gy did not produce an OS benefit compared with the standard dose of 60 Gy, and greater toxicity was still considered to be the main reason [10, 30]. Thus, it is essential to achieve an acceptable balance between therapeutic effect and radiation-related toxicity. Many ongoing studies including RTOG 1106 are focused on delivering high dose radiotherapy while limiting normal tissue doses. To our best knowledge, this study is the first study on individual isotoxic dose escalation in CCRT for patients with unresectable stage III NSCLC based on V20 with new radiotherapy approaches (including PET-CT, SPECT, shrinking field and LCAHF IMRT), particularly with an over 9 years follow up. Besides, the recommended value of V20 in isotoxic radiotherapy of CCRT for patients with LANSCLC was first reported.

Our results indicated that dose escalation in CCRT for patients with LANSCLC and the protocol we used were feasible. The conclusion of RTOG 0617 might imply that it was not reasonable to give all patients a radiation dose enhancement to a particular dose. Isotoxic radiotherapy, which take into consideration the variability of tumor size/volume, anatomical location, stage and normal tissue constraints, is a novel concept of personalised radiotherapy treatment allowing the individualised administration of radiotherapy dose based on predefined normal tissue constraints. This approach ensures the delivery of the maximum achievable biological equivalent dose for each patient whilst ensuring the safety of the OARs. Van et al reported individualised dose escalation with hyperfractionated accelerated 3D CRT was achievable and associated with mild toxicity in a prospective study of 137 patients with medically inoperable stage III NSCLC treated with CCRT [31]. A median OS of 25.0 months was reported in their study, which was the same as ours. A median PFS of 14.0 months in their study was slightly longer than that in ours (13.0 months). The incidence of severe acute toxicity (≥ grade 3) in our study (25.0%) was lower than that in theirs (35.8%), while the incidences of severe late toxicity (≥ grade 3) were similar (7.5% vs. 7.3%, respectively). Obviously, higher incidences of severe toxicity (≥ grade 3) were reported by RTOG 0617: 165 (76%) of 217 patients in the standard-dose group and 163 (79%) of 207 in the high-dose group.

The conventional approach could result in the under-treatment of patients who could potentially receive higher doses of radiation. Our research demonstrated that, on the premise of compliance with OARs dose constraints, patients of LANSCLC accepted CCRT with higher TTD had significant OS and LRFS benifits. In our study, the mean TTD was increased from 59.4 Gy in SD to 71.3 Gy in HD, but no statistically significant difference was found in overall severe toxicity (*P* = 0.488), or in severe pulmonary toxicity (*P* = 1.00) between the two groups. Furthermore, it was worth noting that LRFS was better with HD than SD (*P* = 0.007), and OS was found to be better with HD and an early stage. Otherwise, no significant correlation was found between stage and TTD (*P* = 1.000). We concluded that dose escalation improved LRFS rates, further to OS rates. Our results agreed with those of previous studies, in which improved survival was achieved in patients who received higher total dose with acceptable toxic effects [32, 33]. The median OS of HD group in our research (27.0 months) was longer than that of high-dose group (20.3 months) in RTOG 0617, but slightly shorter than that of standard-dose group (28.7 months) in the same trial, one possible reason was the potential improvements in clinical care. The median OS of SD group in our study (16.0 months) was shorter than that of standard-dose group (28.7 months) in RTOG 0617, apart from the small sample size, the under-treatment of patients might be another reason [34]. Patients of stage III NSCLC were variable because of the small sample size, optimal dose margins could not be found for patients of different subgroups. Whether there were optimal dose margins in isotoxic radiotherapy for different patients subgroups need further clinical trials.

New approaches played a critical role in our research. Advanced technologies including PET-CT, SPECT lung perfusion and IMRT were used. Firstly, several studies [35–38] on the impact of PET on treatment planning for NSCLC suggested an overall improvement in target volume delineation. Radiotherapy adaptive to tumor shrinkage determined by repeated PET-CT after 40 Gy during treatment course might be feasible to spare more normal tissues [22]. Secondly, results have demonstrated that, incorporation of SPECT functional information into conformal radiotherapy planning can allow reduction 3%-17% in the volume of the bilateral functional lung (FL) receiving ≥ 20 Gy, and particularly, where discrete nonfunctional regions of significant size are detected [17, 19]. Thirdly, hyperfractionated and/or accelerated radiotherapy showed an absolute 5-year survival benefit of 2.5% over conventional fractionation [39]. Fourthly, the combination of SPECT imaging data with IMRT techniques had shown the potential to improve FL avoidance when compared to SPECT-based conformal plans [20].

V20 is commonly used as a predictor of radiation pneumonitis. To date, very few studies have investigated the MTV in treatment regimens with concomitant chemotherapy and isotoxic radiotherapy. In this study, we concluded that, for patients meet the enrolled criteria, 37% was the MTV in this treatment regimen and we recommended 35% as the appropriate value for V20 in isotoxic radiotherapy with the help of PET-CT and SPECT.

There are several limitations in this study. Firstly, the relatively small sample size is liable to compromise the generalizability of the findings. Secondly, patient selection is unavoidable in single-institution studies. Thus, larger prospective multicenter studies are necessary. Thirdly, the fact that the efficacy of pemetrexed in CCRT is superior in patients with nonsquamous histology was not known when the study was designed, and all patients accepted the same chemotherapy regimen consisted of vinorelbine and cisplatin. Univariate analysis showed no difference in survival among patients with different pathology (Table 3).

In conclusion, long-term outcomes of our study indicate that individual isotoxic dose-escalated CCRT in unresectable stage III NSCLC patients based on V20 and new radiotherapy approaches is feasible and effective. In the future, the radiation dose escalation for these patients should be focused on toxicity control and advanced technology application.

## MATERIALS AND METHODS

### Patients

Consecutive patients treated at our institution from March 2006 to May 2009 were enrolled for this study. The eligibility criteria were as follows: histologically and/or cytologically diagnosed unresectable stage IIIa or IIIb NSCLC with measurable lesions (according to the cancer staging manual sixth edition of American Joint Committee on Cancer); age of 18 years or older; Eastern Cooperative Oncology Group (ECOG) performance status of 0 or 1; weight loss of 10% or less during the 3 months before the diagnosis; and no history of previous radiotherapy and/or chemotherapy. Patients were also required to undergo brain magnetic resonance imaging (MRI) or CT to rule out asymptomatic brain metastasis at the start of the study. Patients with malignant pleural effusion and/or contralateral hilar node involvement were not eligible. All patients underwent PET-CT simulations and SPECT lung perfusion scans in the same treatment position. All patients signed the written informed consent before the treatment.

### Dose escalation protocol

Isotoxic radiotherapy was based on strict normal tissue dose limits, and the dose-volume constraints on OARs used for plan optimization were as follows: esophagus D_max_ ≤ 75 Gy, spinal cord D_max_ ≤ 50 Gy, heart V65 ≤ 33% (the volume of the whole heart receiving ≥ 65 Gy) and V45 ≤ 67%, hepatic V35 ≤ 50%, and gastric D_max_ ≤ 50 Gy. The maximum tolerated lungs V20 was explored. The acute and chronic toxicity profile of V20 had already been assessed. Patients were divided into cohorts of eight patients in each. Patients with a V20 of 27% as a base level were entered in the first cohort [40]. From the second cohort, the V20 was further increased to 30%, 33%, 35%, and 37%, respectively. The criterion for cessation of dose escalation was defined as 25% of patients or above (i.e., ≥ 2 patients) experienced dose-limiting toxicities (DLTs). We declared this V20 level as the MTV. DLTs were defined as Grade 3 or 4 RTP and grade 4 RTE, according to the Common Terminology Criteria for Adverse Events (CTCAE) v3.0. Patients with TTD over 66 Gy would be assigned to the HD group, while the others to the SD one [41]. Estimation algorithm was used to sum up V20 of two phases radiotherapy (details were described below). Total dose for the first phase of the radiotherapy was defined as D1, total dose for the second phase was defined as D2. Plans for two phases (plan 1 and plan 2) were evaluated according to the total dose of D1+D2 by treatment planning system, and V20 of two plans were acquired respectively (V20^1^ and V20^2^). The final V20 was calculated according to the following formula: V20^1^×D1/(D1+D2)+ V20^2^×D2/(D1+D2).

### Treatment

All patients were assigned to receive concurrent administration of chemotherapy and LCAHF IMRT. In the first phase of the radiotherapy, radiation was administered at a total dose of 40 Gy over a 4-week period, with daily fractions of 2 Gy on consecutive weekdays. In the second phase, hyperfractionation radiotherapy was administered with 1.4 Gy/fraction, twice daily with a minimum interval of 6 h, 10 fractions a week. The total radiation dose was given individualized according to the normal tissue dose limits and V20 of lungs for each cohort.

The gross target volume (GTV), which encompassed only the radiologically visible tumor, was delineated according to the FDG PET-CT images, and elective nodal radiotherapy was not performed. The GTV was delineated and agreed upon by two radiation oncologists and a radiologist on all CT and PET images. The clinical target volume (CTV) was GTV plus a 5- or 7-mm margin in all directions for squamous cell carcinoma and adenocarcinoma [42], respectively. The planning target volume (PTV) was defined as the CTV plus a 5-mm margin in all directions. For involved lymph nodes, an 8-mm margin was added to achieve the PTV. The heart, spinal cord, and esophagus were delineated by the same radiation oncologists. PET-CT was performed again after 40 Gy to shrink radiation fields, and GTV for late course IMRT was the residual visible disease.

SPECT lung perfusion images were co-registered manually with PET-CT images using the ADAC Pinnacle^3^ version 7.4f planning system (ADAC Inc, CA, USA) and were used to define the FL and nonfunctional lung (NFL). FL refers to the region with 30% or more maximum radioactive counts, and the residual region was defined as the NFL [43]. Then, SPECT lung perfusion images were classified into 4 grades by comparing SPECT lung perfusion deficits with areas of radiological abnormality: grade 0 referred to no lung perfusion deficit; grade 1 referred to an area of NFL less than the size of 1 pulmonary lobe; grade 3 referred to an area of NFL exceeding the size of 1 lateral lung; and grade 2 was between grades 1 and 3.

IMRT plans were optimized to minimize the volumes of FL exposed to radiation based on SPECT lung perfusion imaging. During IMRT plans optimizing, beam weights were optimized by minimizing 3 different lung parameters in addition to constraints for the PTV. These lung parameters were the mean functional lung dose, the relative volume of FL receiving ≥ 20 Gy, and the volume of FL receiving ≥ 30 Gy.

Two cycles concurrent chemotherapeutic agents were cisplatin 25 mg/m^2^ on days 1-3 and vinorelbine 25 mg/m^2^ on days 1 and 8. After completion of concurrent chemotherapy and radiotherapy, an additional 2 cycles of vinorelbine and cisplatin chemotherapy were administered. Patients were allowed to receive full supportive care and symptomatic treatments during radiotherapy.

### Follow up, toxicity and response evaluation

Toxicity evaluations, including those for the hematological system, esophagus, gastrointestinal tract, and pulmonary system, were performed weekly during treatment. Patients were followed up every 2 months during the first 2 years after completion of treatments, every 6 months during years 2-5, and annually over 5 years. Evaluations included physical examination, routine blood work, and thoracic CT scan. Brain MRI and abdomen ultrasound were done every 6 months for the first 2 years, and then once a year. Additional tests were ordered whenever there was any indication from results of examinations. Follow-up time was defined as from the end of the treatment to the patients’ death or last follow-up time. Patients were scored for toxicity and evaluated for tumor response according to CT scans. Tumor responses were evaluated according to the Response Evaluation Criteria in Solid Tumors (RECIST) [44]. Toxicity was graded according to the CTCAE v3.0. OS was defined as time from diagnosis till death or last follow-up time, and PFS was defined as the time from diagnosis until first clinical event (local or distant progression, death from any cause or last follow-up time).

### Statistical design and analysis

Descriptive statistics were generated for toxicity and response. The Kaplan–Meier method was used for univariate survival analysis (log-rank test) testing the following variables: tumor stage, TTD, age, pathology, lung V20, lung perfusion deficit, short-term effects, toxicity, maximal standard uptake value (SUVmax) and SUVmean. SUVs were abstracted from PET-CT before treatment. Grouping criteria were listed in Table 4. The Cox proportional hazards model was used for multivariate analysis. Comparisons between SD and HD, IIIa and IIIb groups were evaluated using the Chi-square test, Fisher's exact test. All statistical analyses were performed using SPSS 22.0 (IBM Corp, NY, USA). A *P* value of < 0.05 was considered significant.

**Table 4:** Fators grouping criteria in Kaplan–Meier method

Factors	Assignment instructions					
Tumor stage	III a = 1	III b = 2			
TTD (Gy)	50-66 = 1	> 66 = 2			
Age (years)	≤60 = 1	61-70 = 2	>70 = 3		
Pathology	SCC = 1	AC = 2	LCC = 3		
V20 (%)	27 = 1	30 = 2	33 = 3	35 = 4	37 = 5
LPD (grade)	0 = 0	1 = 1	2 = 2	3 = 3	
Short-term effects	CR+PR = 1	SD = 0			
Toxicity	0-2 grade = 0	≥3 grade = 1			
SUVmax	≤10 = 1	>10, ≤20 = 2	>20 = 3		
SUVmean	≤4 = 1	>4, ≤6 = 2	>6 = 3		

Abbreviations: TTD= total tumor dose, SCC= squamous carcinoma, AC= adenocarcinoma, LCC= large cell carcinoma, V20= volume of the whole lung receiving ≥ 20 Gy, LPD= lung perfusion deficit, CR= complete response, PR= partial response, SD= stable disease, SUVmax= maximal standard uptake value, SUVmean= mean standard uptake value.
